# Supplementary figures and images for: A Putative long-range RNA-RNA interaction between ORF8 and Spike of SARS-CoV-2
Source: PLoS One. 2022 Sep 1;17(9):e0260331. doi: 10.1371/journal.pone.0260331 (PMC9436084; doi:10.1371/journal.pone.0260331)

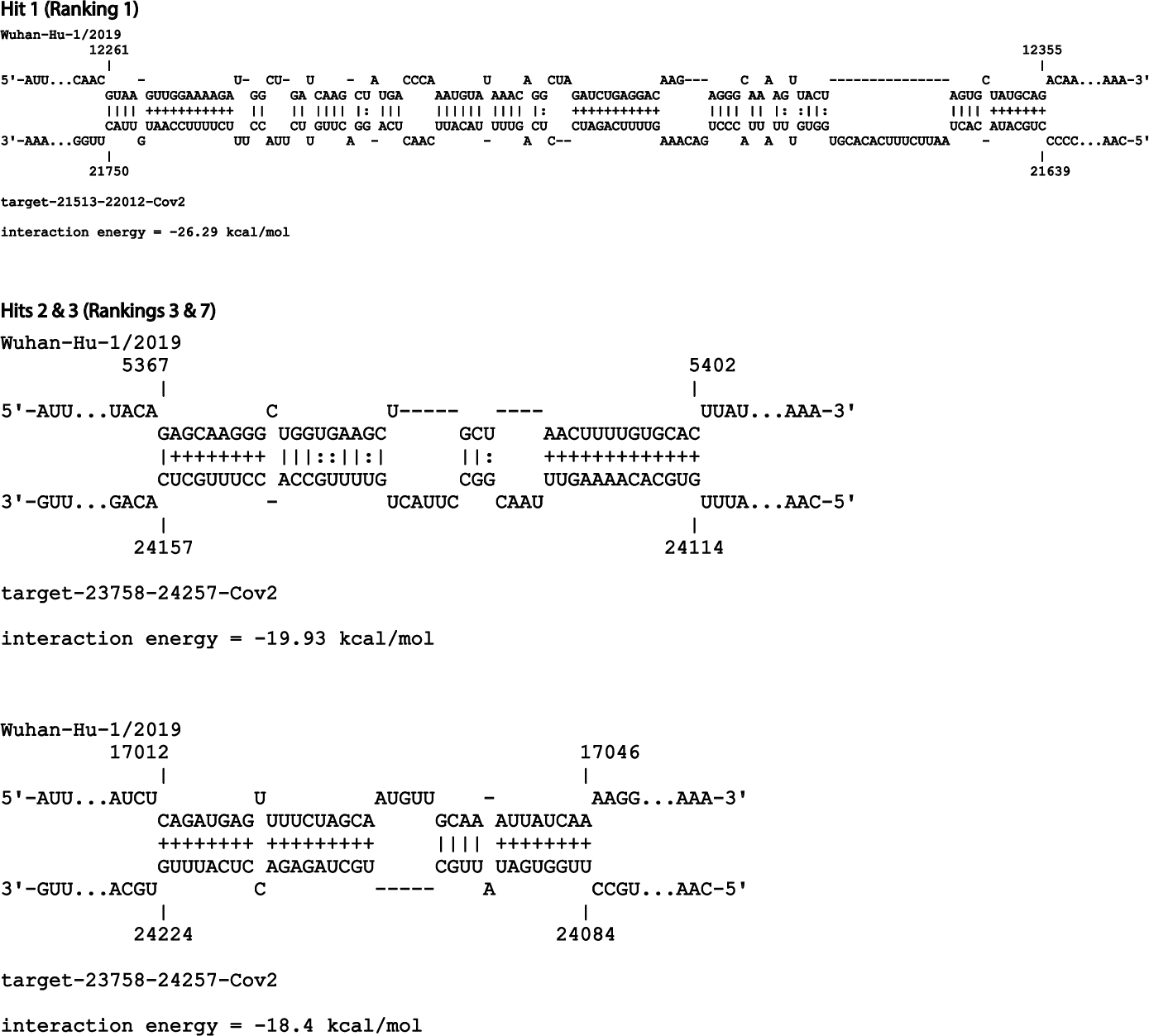

Supplement: S1 Fig — Corresponding ranking of the hits are also included. Generalized linear model was used to rank hits with highest interaction energy relative to interaction length (Table 1). (TIF) [file pone.0260331.s001.tif]

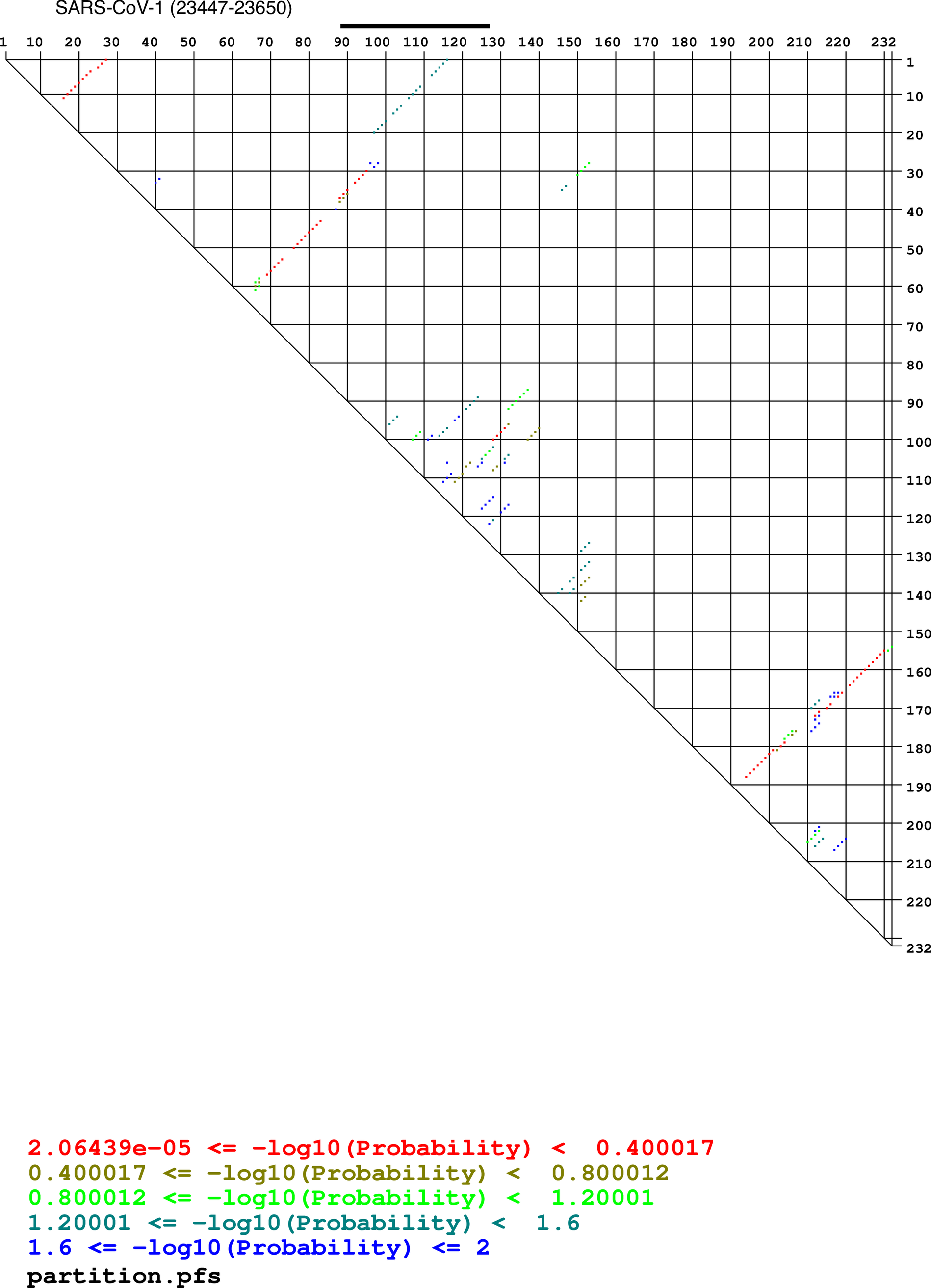

Supplement: S2 Fig — Probabilities were calculated using McCaskill’s partition function [51, 52]. Coordinates: SARS-CoV-1 (23447–23650 Spike). SARS-CoV-2 Spike/ORF8 region (23660–23703) was mapped on SARS-CoV-1, with coordinates: SARS-CoV-1 (23534–23575 Spike) and extended on both directions by 100nt. The resulting region is highlighted as a black bar. (TIF) [file pone.0260331.s002.tif]

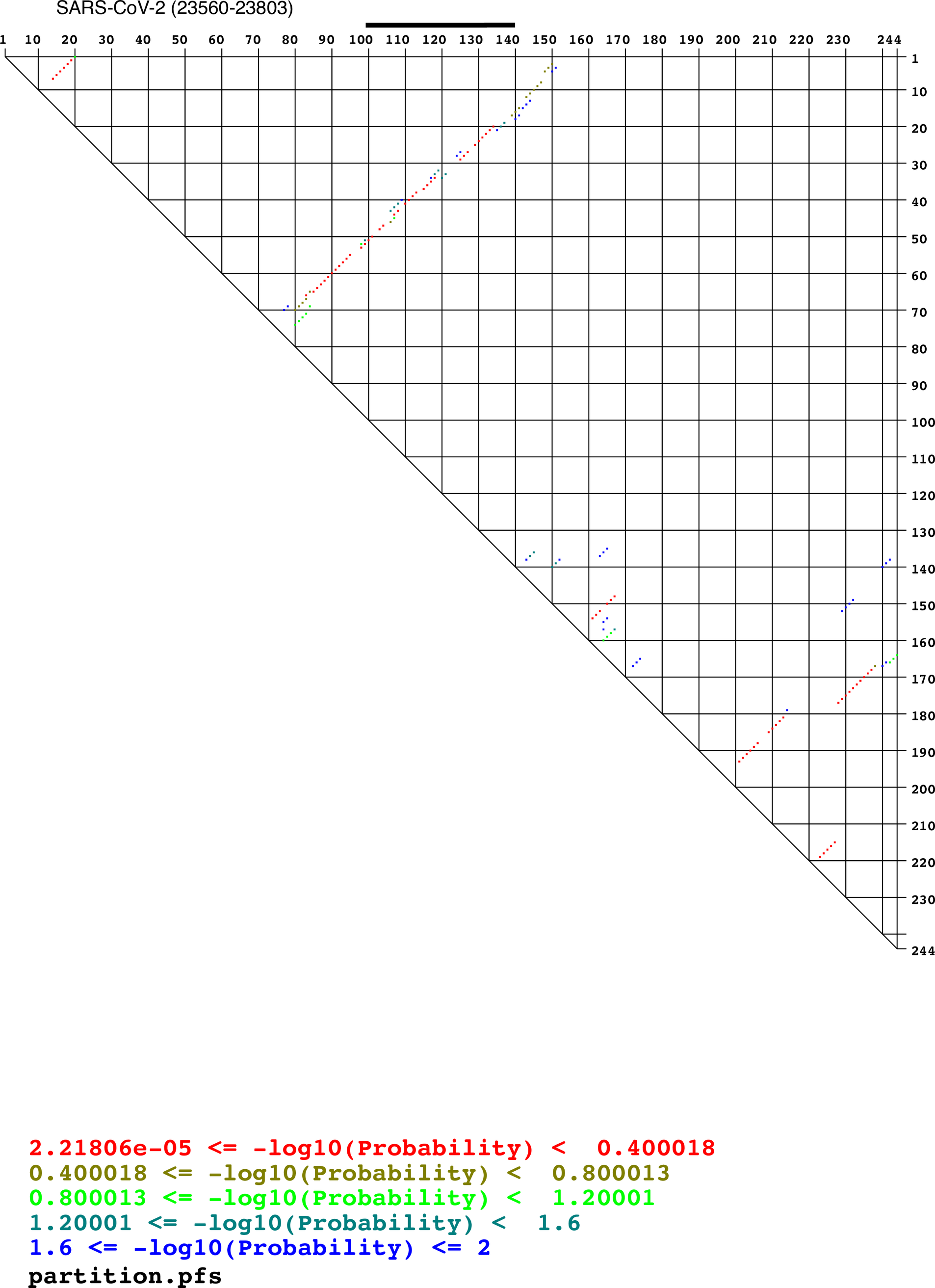

Supplement: S3 Fig — Probabilities were calculated using McCaskill’s partition function [51, 52]. Coordinates: SARS-CoV-2 (23560–23803 Spike). The Spike/ORF8 region (23660–23703), shown as a black bar was extended on both directions by 100nt. (TIF) [file pone.0260331.s003.tif]
