# Supplementary material for: A Putative long-range RNA-RNA interaction between ORF8 and Spike of SARS-CoV-2
Source: PLoS One. 2022 Sep 1;17(9):e0260331. doi: 10.1371/journal.pone.0260331 (PMC9436084; doi:10.1371/journal.pone.0260331)
Supplement: Attachment — Submitted filename: Response to reviewers-final-R2-SCJ-edits.pdf [file pone.0260331.s007.pdf]

Response to Reviewer's Comments

Reviewer #1: # Review for PONE-D-21-35236-R1

Evidence for a long-range RNA-RNA interaction between ORF8 and Spike of SARS-CoV-2

Okiemute B. Omoru, Filipe Pereira, Sarath Chandra Janga, Amirhossein Manzourolajdad

Summary

In short, the authors want to identify putative long-range RRIs within the SARS-CoV-2 genome that have high chance to be virus specific. To this end, they do a comparative investigation with SARS-CoV-1. Since there is a SARS-CoV-2-specific high-GC insert in Spike, the authors focus on this gene. Using a sliding window approach, putative interaction sites of Spike with other genomic regions are identified for both viruses. Since only SARS-CoV-2 shows a stable RRI with ORF8, the authors focus on the support of one of the predictions using structure prediction, base pair probabilities and (co-)variation analyses.

General remarks

The revised work has positively sharpened the focus of the manuscript and provides more motivation for the whole endeavor. While improved, it still shows flaws and is still, in my opinion, of limited interest.

My major points are:

(*) There is no evidence anywhere that the identified RRIs are true "long-range" interactions!
The best one can speak of are "putative long-range" interactions, since (a) the whole identification and prediction is *fragment-based*, *in-silico* and without evidence that the full genome forms the interaction. It is also quite likely that the interaction is formed but only by (m)RNA fragments such that the whole hypothesis of the manuscript would be lost...

This is nowhere discussed within the manuscript!
Furthermore, both title and main contributions of the article need to be rephrased that way..

Response: We thank the reviewer for these suggestions and have now revised the title and discussion to reflect this input. The main hypothesis is the predicted Spike-ORF8 interaction. Various mechanistic speculations as discussed by the reviewer are mentioned in the discussion. The in-silico fragment-based nature of our approach is also emphasized in Abstract, Introduction, and Discussion. The main contribution of the work is further clarified. We thank the reviewer for encouraging us to clarify our main finding regarding integration of thermodynamic-based modeling and mutation patterns to identify the core sub-interacting region in the Spike-ORF8 prediction.

(*) The presented Pseudoknot structure prediction is neither local nor in line with the other used tools. While it is hard to impossible to study or compare the dot-bracket-reported PK structures (Table 3), I find their presentation of no use and eventually wrong in the used context. First, the ProbKnot tool does a GLOBAL mfe prediction, with the limitations I discussed in the last review. Thus to infer local structure information from single mfes is, in my opinion, optimistic and wrong. Furthermore, all other used models

within the study (IntaRNA and bp-prob computation) are based on nested structure models.. Thus, the base pair probs discussed along with the PK are using a different energy AND structure model and are thus hard to compare and a wrong intuition is triggered by the current text layout (namely that bp probs and PK structure are related).

Why using it at all? In the end, only a single minor crossing helix (5bp) is found and none for SARS-CoV-1. I doubt the relevance of this observation. Even more so while the authors have no mechanistic or whatsoever explanation or discussion of the observation (beside that it is observed).

To sanatize the course of the manuscript, I recommend to drop the PK part.

Response: As recommended by the reviewer, we have now removed the PK part from the manuscript to improve the clarity and flow of the manuscript.

(*) Inconsistencies within the manuscript

- the Methods section still lists tons of tools that are not used in the current version

Response: We have now removed the listing of tools in the methods section which are not used in the current version of the manuscript.

- the manuscript still refers to bifold predictions that are not present

Response: We have now removed the bifold predictions from the manuscript.

- the 2nd and 4th paragraph of the introduction are mainly redundant

Response: Thank you for this suggestion. We have now reduced the redundancy in these paragraphs of the introduction.

- the RRI visualizations (taken from IntaRNA) are using local fragment indices for Spike rather than the genomic positions, which makes it hard to follow and map the information. Just edit! (both in Fig-2 and supplement)

Response: We appreciate the reviewer pointing to this inconsistency and have now edited the figure 2 to reflect the genomic positions so that it is easy to map and follow the information across the study.

(*) Missing rationals

- the reason why the authors investigate the Spike-ORF8 RRI is only given within the discussion, and one is lost wondering in the result section

Response: To improve the flow and logic for investigating Spike-ORF8 RRI in this study, we have now included a transition in the result section right before the Spike-ORF8 section providing a rational for choosing these regions for performing RRI analysis in this study.

- the use and interpretation of the AIC is no where to find and it stays unclear if the reported values are good or bad or how to interprete at all..

Response: We have now removed AIC from the main text. As a measure of model fitness, we used the significant factor of the Length parameter instead. (Pr(>|t|) for length is reported in Table 1 caption along with other details of the model used. We have also explained details regarding model derivation.

(*) Missing data

- the genome versions are not given (important since the reference genomes are undergoing some changes)

Response: We have now included the specific version of the genomes that were used in the study.

- the supplement lists the whole set of 2 million genome IDs but not the 200k used for the analyses

Response: As suggested by the reviewer, we have now included the genome sequence IDs for the 200K genomes that were used in the analyses.

- how was the linear energy model derived? tool? library? handwritten?
Response: The linear energy model was generated in R statistical package, and we now have explained it in the manuscript.

(*) Overstating seed base pairs of RRIs

The "+" annotated bps in IntaRNA outputs are from stable subinteractions (of a used/default defined length, typically 7bp). Thus, these so called seed interactions are (in itself) stable enough to form (i.e. typically in unstructured regions) and thus likely to be starting points of the full RRI formation. Since an interaction can cover multiple such regions, all respective bps are annotated.

Currently, the manuscript describes these basepairs as "those that pair earlier than other base pairs", which is not true but again just a hypothesis.

Respective formulations should be amended respectively.

Response: We have now changed multiple places in the manuscript to reflect the above interpretation recommended by the reviewer. We refrained from speculations regarding the above results.

(*) Artifacts from subopt-limit

Since the authors limited the predicted suboptimals to 5 per fragment, the interaction atlas presented in Fig-

1 is limited too. It could be that certain regions could interact with even more regions, which just dont pop up due to the hard "top-5" limit.

While this is no big drawback, it needs discussion. Even more so since the lack of predicted RRIs is a central point of discussion within both the result as well as discussion section!

Response: As suggested by the reviewer, we have now elaborated the discussion to reflect on the possibility that there could be additional RRIs which may have escaped our limit of top 5 hits but still could be biologically interesting.

(*) Suggestion: alpha via p-values

Just a suggestion that could improve the presentation. Given a large amount of genome-wide predictions (as done here) it is possible to estimate p-values for the energy scores (as presented on the IntaRNA webserver). The IntaRNA package even provides a respective script for computation.

The p-values could be used to set the alpha channel of the arcs within the circle plots to highlight highly probable RRIs and to distinguish them from weaker ones.

Currently, it is hard to say what interactions are stable. Even more so, since there is no general energy cut-off etc.
Response: We agree that p-values and/or setting cut-off are also good methods for producing meaningful results. In this work, however, we had decided to use an alternative approach for ranking predictions, which is the residual values of our model as discussed in results section.

(*) Fig 4 not interpretable in printed version (and hard in pdf)
The dot plots are so small that dots are hard to spot or interprete/compare in print.

But even in pdf this is hard since only a pixel graphics is provided that cannot be zoomed without getting pixelated.

I suggest to move both figures in vector graphics format (PDF) to the supplement in full page width each to allow for detailed investigation.

The authors could present a respective cutout for the main manuscript if needed.

Response: We have now made a concerted effort to significantly increase the resolution of Figure 4 for better readability. However, please note that PLoS One submission system often decreases the resolution of submitted figures for peer review purposes to generate less heavy files for reviewers and it may be possible that this has resulted in down resolution. Nevertheless, as suggested we have now also included the figure as supplementary file too.

(*) Minor issues that caught the eye

- the text uses "Orf.." instead of "ORF.."
- Table 1 (and the supplement table) do not use "ORF8" but rather just "8" etc.
- Table 1 shows a red highlight not discussed in its caption

- "11th top hit within a total of 66" .. what 66? or is about the 69?
- "segments each corresponding to a particular viral strain" .. nope, each corresponds to a full genome sequencing (i.e. sample) but not necessarily strain!
- Table 2: lines Spike 23679-80 should be bold too
- it is not discussed that the CaCoFold predictions (Table 2 + Fig 3) miss the left-most RRI part from Fig-2, thus, rendering that RRI part less likely
- Fig-3 seems to be of low quality
- no vector graphics.. zoom in provides pixel art ..
- "contains five pseudoknots" .. NO, only ONE KNOT but "5 crossing base pairs". A BIG difference!
- Fig-4: it would be helpful to state in the caption that most likely base pairs are colored in red (for non-math readers)
- the text often states "SARS-CoV" instead of "SARS-CoV-1"
- Fig-4 caption "using the partition function" is a useless comment. better name the used tool.
- Fig-4 "shown in bold" .. better use "highlighted with a black bar". You can also annotate the same region on the y-axis (same coordinates) and draw horizontal/vertical lines at the respective bar ends to guide the eye to the important corridors within the plot
- "as well as well"
- it would be interesting to relate the S1 hairpin with the dot plot or annotate it(s position) within
- the supplementary figure needs a caption or the figure has to be extended to be self-explaining (what is relating to what and where)

Response: We sincerely thank the reviewer for these suggestions and have now made every effort to address all these minor issues to significantly clean up the manuscript.

(*) Carving out the core RRI based on the variation and stability investigation

Eventually, I think the authors miss a central outcome of the study or do not present it as such. While all the "long-range" part and the hopeful hypothesis of its impact on virulence, regulation etc. is quite speculative, the authors miss to highlight that the integration of RRI prediction and variation analyses strongly identifies the core of the putate Spike-ORF8 interaction. Namely the seed-region stretch 23679-23690 (Spike). The left part of Fig-2 is not predicted in Fig-3 and no variation is seen in this area.

Thus, one can conclude that the true RRI part (or at least the most likely part) is defined by that region and that it might be relevant (but maybe not exclusively for the RRI) since it is not mutated in both genes.

Why is it that most core conclusions of that manuscript are by reviewers?

Response: We appreciate the input from the reviewer, but we want to emphasize that study's main goal was to identify the core RRIs in the Spike-ORF8 regions as the main contribution. Co-variation analysis was initially anticipated to be an independent means for understanding the functional meaning and evolutionary conservation of these inferred associations.
