# Supplementary material for: A Putative long-range RNA-RNA interaction between ORF8 and Spike of SARS-CoV-2
Source: PLoS One. 2022 Sep 1;17(9):e0260331. doi: 10.1371/journal.pone.0260331 (PMC9436084; doi:10.1371/journal.pone.0260331)
Supplement: Attachment — Submitted filename: Response to reviewers-final.docx [file pone.0260331.s006.docx]

**# Review for PONE-D-21-35236**

**Response to referees:**

We are grateful to both the reviewers for their enthusiastic feedback and constructive criticism on our manuscript. We are also thankful for the helpful suggestions in improving the different sections. We have now incorporated the suggestions provided by the reviewers and hope that the revised version of the manuscript will now meet the expectations of the referees. In particular, the revised manuscript has been re-organized to address some of the major points raised by both reviewers regarding the underlying assumption of the work. In addition, we considered a bigger and a more updated dataset for our population analysis (around 200’000 rather than previously 27’000 sequences). Below are the responses to the raised queries:

**Point-by-point response to reviewer’s concerns:**

**Reviewer #1:** 
\# General remarks

I have couple of central issues with this manuscript in its current form.

\## (1) 12 nt insert irrelevant and obscures the focus and informative value

Beside the definition of the genomic subregion of interest, the 12 nt insert is eventually unimportant for any statement supported by the study (beside observing that S1 stem loop is stable with and without the insert).
The authors write themselves "The contribution of the 12-nt insert [...] is still unclear".

Thus, I have to ask: why then using the 12-nt fragment as such a rigid anchor for the study? The authors use about 2/3 of the Spike gene somewhat at random (based on the 12nt and its structure formation) instead of the whole gene! The rational for taking the whole could be the same, i.e. "12-nt insertion is inside", but now the SARS-CoV-1 subregion is clearly defined (as the related full gene sequence) and not based on the mapping of a substructure region...

Using just a subregion of the gene
(a) causes problems with local structure prediction AT LEAST at the ends of the regions if not overall,
(b) has thus implications on RRI prediction, since IntaRNA incorporates the accessibility of interacting subregions, which is based on local structure prediction, and
(c) restricts the insights of the study to a hypothetical local fragment rather than a valid mRNA-like molecule.

**Response:** We agree with the reviewer’s concern about the 12-nt-insert bias and appreciate the pertinent feedback. Hence, as suggested above, the rationale presented in the revised manuscript is now based on investigating the complete Spike region, as opposed to being exclusive to surrounding regions of the 12-nt polybasic insert. In the previous version of submission, the introduction and a great part of results (Figures 1 and 2) were based on the 12-nt polybasic insertion narrative, whereas the current version is based on the suggested rationale. Our hypothesis is now to see *if SARS-CoV-2 Spike region has evolved on RNA level compared to SARS-CoV-1*. The emergence of the GC-rich 12-nt polybasic insertion in SARS-CoV-2 *Spike*, is presented in the introduction as the background and motivation to drive a detailed analysis to understand the RNA structural evolution in *Spike*. The newly presented work (Figure 1 and Table 1) is the result of scanning the complete Spike region and is not biased to the 12-nt. We have also significantly revised and improved the introduction as well as most of the results sections, considering the comments from both the reviewers.

\## (2) mfe structures based on global folding are no local structure signal

Using minimum free energy (mfe) estimates based on GLOBAL structure prediction (i.e. allowing base pairing over the range of the RNA) and respective structure plots to discuss local structure formation is inappropriate. The mfe is only ONE possible stable structure and strongly depends on the underlying thermodynamic model. Mfe prediction gets decreasingly reliable (in its details) the longer the RNA. Thus, instead of individual base pairing (of the presented mfe structure), LOCAL base pair probabilities (eg visualized via dot plots) are a much sounder tool to investigate local structure formation of RNAs or genomic regions. And if the details of base pairing of of no importance, even local unpaired probabilities (related to the accessibility values used by IntaRNA), are simple tool to identify locally (un)structured regions etc. without detailing base pairing.
When presenting mfe predictions of different tools (Fig-1) that are based more or less on the same thermodynamic energy model, no further insight is gained...

Furthermore, structure representations (Fig-1, Fig-4) are

-) so small that they are not readable in print
-) due to the latter, coloring is completely lost in print
-) eventually unimportant and not discussed in details for the RRI prediction

**Response:** We thank the reviewer for this insightful comment and agree with the reviewer’s concern that MFE-based structural specifics are not critical and can be omitted. All figures relating to RNA structure prediction are omitted from the revised manuscript as they did not carry critical information with regards to the main point of the manuscript. As suggested by the reviewer, visualizations via dot plots are now used instead, to study local structures (Figure 4). Please also note that our arguments and conclusions are based on the inferences about base-pairing probabilities rather than MFE-based base pair formation (See Discussion Paragraph 7, Locally stable RNA base pairs …). We have also improved the quality and layout the figures so that they are easily readable and interpretable.

\## (3) There is no covariation as far as I can see...

Please check Fig-1a of

<https://academic.oup.com/bioinformatics/article/36/10/3072/5729989>
the only thing I observe in the provided data (Fig-3) is VARIATION, i.e. only one side of the base pair shows variants...

The whole covariation part has several flaws..

(a) the authors use the concatenated interacting subsequences as an input to the CO-FOLDING BASED R-scape software... This is terribly wrong, since no LINKER sequence spacing the two FRAGMENTS is used. Without it, the helix close to the interaction that is close to the point of concatenation cannot be formed due to STERICAL reasons respected by the structure prediction software... Thus, the co-variation study misses eventually 5 base pairs and even more important, the Spike region is ONE TOO SHORT! missing the final interacting U...?!?

If done that way, ie. using the co-folding based approach, one has to insert a linker region of at least 3-5 nt, eg. poly-A or if possible poly-N, to allow for enough flexibility that the concatenated ends of the sequences can form a structure. Or why not extending the subregion range by 5nt in both directions? would be simple and avoid this central error..

**Response:** We agree with the reviewer about this error and have taken measures to apply the linker in the corresponding analysis. One of the suggestions was to extent the actual sequence by 5nt on both ends (totalling 20nt for each pair of segments). Original segments were extended from 23660-23-703 and 28025-28060 to 23655-23-708 and 28020-28065. Section Materials and Methods, subsection *Compensatory Mutations Analysis of Long-range RNA-RNA interactions*, Paragraph 2 contains the details about the modification in this revision.

(b) who does the counts in Fig-3b relate to the substitution count in Table-1? The authors refer to the manual of R-scape, which is in no way a proper scientific explanation, even more so since these counts are central for selecting pairs and position within the argumentation of the authors..

**Response:** This point has now been disambiguated. Actual counts of mutations are explicitly extracted from data and presented in Figure 3 for the top four cases.

(c) the substitution counts incorporate AMBIGOUS nucleotides (Fig-3b)... ?!? What about sequencing errors etc. as a source of such ambiguity? For other parts of the study such subsequences were excluded, why not here?

**Response:** As suggested, we removed any ambiguity from our dataset. All remaining symbols are strictly {A,G,C,U} at all steps. Mutation counts in Figure 3 also reflect this change.

(d) the authors MISS an important point: the IntaRNA predictions are annotated with based pairs that are likely to be formed first, the seed base pairs represented by "+" within the plot. These base pairs seem to correlate with LOW substitution scores, which I would find a supportive and good sign! That is low sequence variation in the important sub-RRI!

**Response:** We thank the reviewer for bringing this observation to our attention. The “supportive and good sign…low sequence variation in the important sub-RRI” mentioned by the reviewer remains consistent even in our newer and bigger choice of dataset. Comparing Figures 2 and “power” column of Table 2, we can clearly see that the reviewer’s observations holds and that the more critical bonds (shown by ‘+’ in Figure 2) have lower variation (0.0 power). The above point has been mentioned in Discussion (paragraph 4, *The population of SARS-CoV-2 sequences …*) as one of the two main pieces of evidence for the Spike-ORF8 RNA-RNA interaction.

Thus, an investigation and relation of VARIATION with RRI formation would be more appropriate than a co-variation study that shows there is no co-variation..

**Response:** We agree with the reviewer on no sign of covariation in the data and have explicitly mentioned that in the discussion.

\## (4) Why showing bi-/tri-molecule co-folding results?

I find no rational for nor any additional insights from the bi-/tri-molecule folding part of the manuscript. The description of the assembly of the tri-molecule prediction from bi-molecule graphs is very vague.

Maybe I missed it, but what's the point? Fig-4a shows the same RRI patterns as Fig-2a. The lack of structure relation of Fig-4b and Fig-2b can/will have multiple possible reasons that are not discussed at all..

**Response:** As suggested by reviewer, bi/tri-molecule co-folding results are now eliminated as they were mostly based on speculations and did not have a clear hypothesis. The corresponding figures and discussion was also omitted in the revised version.

If you are interested in co-formation of multiple RRIs, why not using a constraint IntaRNA prediction? Therein you can mark regions that are involved in base pairing (eg. an RRI) and you will get corrected predictions for the rest of the molecule EXCLUDING the ballast of local intra-molecular mfe structure (see (2)). But eventually, that wasn’t even the point, or was it? I am confused by this part..

**Response:** As indicated above, we have now removed the co-formation of multiple RRIs as it is not directly relevant to the core hypothesis of the manuscript and the supporting data and discussion was weak. We thank the reviewer for pointing to this weakness of this section.

\## (5) No suboptimal RRIs investigated/shown/discussed ...

For RRIs, the same holds as discussed for (2). While base pairing probability prediction is much harder for RRI prediction compared to intra-molecular structure prediction, one can relate to suboptimal interactions as a substitute to assess the structural variety. This is needed at multiple parts within this manuscript:

**Response:** As indicated above, for each individual search, four sub-optimal predictions have been included making it a total of 5 predictions. The Supplementary Table 1, column ranking indicates the ranking of each hit. With regards to applying local stability, although the above suggestion of including local stabilities are constraint on the long-range interaction can be informative in terms of validity of the long-range binding. we did not explore this analysis in this work. Our reasoning was that the application of local stability constraints may be a very stringent constraint in initial analyses, especially for complex RNA structural mechanistic, where two regions may be in competition to bind.

(a) the extremely long UTF interaction in Fig-2b is most likely an artifact of the summation-based prediction scheme. Simply spoken: the more base pairs the better until accessibility penalties overweight the gain.

BUT: did you check suboptimal interactions of the region? I would expect much shorter sub-RRIs with similar stability (i.e. energy).

**Response:** We absolutely agree. Sub-optimal interactions, as suggested, were considered in the revised version. Sequence segments of 500nt (with 50nt overlap) were considered by the software, rather than applying the complete Spike gene in only one try. In addition, we used IntaRNA parameters to include top five hits rather than just one optimal hit for each run. Finally, using IntaRNA parameters, we constrained the program to exclude overlapping targets, to avoid repetitive results.

(b) Furthermore, did you check if a similar ORF8 interaction is among the sub-optimals of the SARS-CoV-1 predictions? Since the ORF8 SARS-CoV-2 RRI shows a lower energy that the UTF-RRI, the chance is high that it is "hidden" there...

**Response:** We thank the suggestion of the reviewer and have now investigated this possibility. However, this was not the case and even in the most inclusive sense, no hit between Spike and ORF8 in SARS-CoV-1 ever appeared. (Figure 1, and Supplementary Table 1). We find this to be a meaningful result. The observation that ORF8 appeared only in SARS-CoV-2 analysis, is detailed in the manuscript as one of the main pieces of evidence for the Spike-ORF8 RNA-RNA interaction to be specific to SARS-COV-2.

Although it may be possible to increase our prediction thresholds to generate more than 5 hits and re-investigate this further, but we doubt that this will lead to meaningful results as we already have many short-sequence hits in our results which might already be false positives. Secondly, in order to minimize the effect of length in ranking stability of interacting regions we used a generalized linear model and trained it on the results (Table 1 and Supplementary Table 1). This estimation also addresses the issue of length in the UTF-RRI (UTR-RRI) region raised by the reviewer. Indeed, the Spike-ORF8 interaction was in the top quantile of our results.

**Reviewer #2:**

1. The 12-nt insertion site is at position 23,603-23,614 of the genome. The authors searched for long range interactions of any SARS-CoV-2 region with the region 23,600-24,107 and 23,917-24,118, i.e., only downstream region of the insertion site. Shouldn't equally long stretches upstream of the insertion site be considered as well? It is not clear to me why the present study has focused on the downstream region only.

**Response:** We completely agree with the reviewer suggestion. Hence, in the revised version, we focused on scanning the complete Spike gene which includes not only the upstream of the insert but also 50nt upstream of the start codon of Spike. We have also included 50-nt downstream of the stop codon of Spike.

2. Similar to my previous question, it is not clear why the overlapping regions 23,600-24,107 and 23,917-24,118 were analyzed separately. It will be helpful if the authors can comment on this.

**Response:** In the original submission of this study, the choice of intervals was largely arbitrary. We agree with the reviewer’s comment that this forms a bias, and it is ad hoc. Hence, in the revised analysis we now equally consider all 50nt overlapping segments within Spike and have significantly revised the manuscript’s figures and results section.

3. The developers of IntaRNA have shown that integrating experimentally obtained chemical probing data with IntaRNA can significantly improve RNA-RNA interaction prediction. Since such data is available for SARS-CoV-2, have the authors considered using it in their analysis?

**Response:** Unfortunately, we did not consider the probing data. We agree that experimental data on full-genome of the virus (rather than on the individual sub-genomic RNAs which to our knowledge is currently available in the public domain in the form of SHAPE profiles from Anna Pyle’s lab) would have led to a more reliable long-range prediction analysis.

4. In the second subsection of the Results, the authors report long-range interaction predictions from IntaRNA. Are these all the predictions from IntaRNA or have the authors reported the subset of the most significant predictions? It might be worthwhile to add a Supplementary table listing all the interactions that meet a reasonable significance cutoff.

**Response:** The supplementary Table 1 includes all possible interactions found between Spike and the rest of the genome including very weak and sub-optimal interactions. We agree that these predictions rely on assumptions and more comprehensive experimental verifications are needed to confirm their *in-vivo* validity as suggested by the reviewer. However, high confidence predictions as listed and discussed in our study would form a roadmap for further understanding, interpretation and validation of these theoretically sound predictions.

5. In the paragraph above Table 1, it seems there is a typo in the second sentence. For the region 23,675-28,045, the table shows n=92 while the text says n=2.

**Response:** We apologize for this oversight and have corrected/updated these values.

6. There are many grammatical mistakes scattered throughout the manuscript, which should be corrected, if necessary, using free software such as Grammarly.

**Response:** The revised manuscript has been re-written significantly and now proofread by multiple native English-speaking colleagues.
